# The Extract of *Litsea japonica* Reduced the Development of Diabetic Nephropathy via the Inhibition of Advanced Glycation End Products Accumulation in db/db Mice

**DOI:** 10.1155/2013/769416

**Published:** 2013-05-27

**Authors:** Eunjin Sohn, Junghyun Kim, Chan-Sik Kim, Yun Mi Lee, Kyuhyung Jo, So Dam Shin, Joo Hwan Kim, Jin Sook Kim

**Affiliations:** ^1^Korean Medicine Based Herbal Drug Research Group, Herbal Medicine Research Division, Korea Institute of Oriental Medicine (KIOM), Daejeon 305-811, Republic of Korea; ^2^Department of Life Science, Gachon University, Seongnam, Gyonggi-do 461–701, Republic of Korea

## Abstract

Increasing evidence indicates that advanced glycation end products (AGEs) contribute to the pathogenesis of diabetic nephropathy. The aim of this study was to investigate the protective effect of *L. japonica* extract (LJE) against renal damage in the db/db mouse. LJE (100 or 250 mg/kg per day) was given to diabetic mice for 12 weeks. Body weight, blood glucose levels, glycated hemoglobin (HbA1c) levels, and proteinuria were examined. In *in vitro* assay of the inhibition of AGE formation, immunohistochemical analysis of podocyte loss and AGE accumulations were performed. In 20-week-old db/db mice, severe hyperglycemia developed, and proteinuria was significantly increased. Diabetes induced markedly morphological alterations to the renal glomerular cells. AGE accumulations and podocyte loss were detected in renal glomeruli. LJE treatment significantly reduced proteinuria and AGE accumulations in diabetic mice. Moreover, the loss of nephrin, an important slit diaphragm component in the kidneys, was restored by LJE treatment. Our studies suggest that LJE might be beneficial for the treatment of diabetic nephropathy. The ability of LJE to attenuate proteinuria and podocyte dysfunction may be mediated by the inhibition of AGE accumulation in the context of diabetic nephropathy in db/db mice.

## 1. Introduction

Diabetic nephropathy is one of the most serious complications of diabetes mellitus [1]. Experimental and clinical researches have revealed that the increased accumulation of AGEs plays a significant role in the pathogenesis of diabetic nephropathy [2]. The levels of AGEs are much higher in patients with diabetes [3]. The clinical hallmarks of diabetic nephropathy include progressive proteinuria followed by a gradual decline in renal function, and glomerular mesangial expansion has been identified as a pathological precursor of these clinical changes [4]. Moreover, it has been reported that AGEs induce the apoptosis of cultured murine podocytes. AGEs have been hypothesized to be a potential causative agent of podocyte damage [5].

Podocytes are one of the important components of the filtration barrier and have special cytobiological traits and physiological functions. The injury of podocytes inevitably leads to the occurrence of proteinuria [6]. Clinically, glomerular proteinuria is most commonly observed, and this condition is related to structural and functional abnormalities in the glomerular filtration barrier [7]. In recent years, researchers have not only gained a new understanding of the roles of glomerular endothelial cells and the glomerular basement membrane in the pathogenesis of proteinuria but have also gradually discovered the close relationship between slit diaphragm molecules such as nephrin and the pathogenesis of proteinuria [8].

Some medicinal herbs have been used for the treatment of diabetes and diabetic complications [9, 10]. In the past few years, many herbal extracts have been screened for possible AGE inhibitory effects *in vitro* in our laboratory. *Litsea japonica* (Thunb.) Jussieu is an endemic plant and grows in southern areas of Korea. Sometimes, this plant is also utilized as a vegetable food and an ornamental plant [11]. It was reported that this plant may have anti-inflammatory effects [12, 13]. However, the effects of this plant on diabetes and diabetic complications have not yet been investigated. Therefore, in this study, we investigated the preventive effect of an ethanolic extract of *L. japonica* (LJE) on renal damage in a type 2 diabetes animal model, the db/db mouse.

## 2. Materials and Methods

### 2.1. Animals and Treatment

All experiments were conducted according to the National Institutes of Health (NIH) Guide for the Care and Use of Laboratory Animals and were approved by the Korea Institute of Oriental Medicine Institutional Animal Care and Use Committee. Male C57BL/KsJ db/db mice (db/db) and their age-matched lean littermates (db/+, normal) were purchased from Japan SLC (Shizuoka, Japan). At 7 weeks of age, the db/db mice were randomly assigned into four groups (*n* = 10). LJE was dissolved in vehicle (0.5% w/v carboxyl methylcellulose solution) at the concentration of 5 mg/mL. Three groups of db/db mice received daily gastric gavage with LJE (100 and 250 mg/kg) or metformin (MET, 350 mg/kg), and the other group was given the same amount of vehicle via gavage for 12 weeks. Nondiabetic littermates received the same vehicle treatment. The blood glucose level and body weight were monitored consecutively.

### 2.2. Preparation of *L. japonica* Extract (LJE)

The aerial parts of *L. japonica* were collected from the Jejudo, an island located in the south of Korea, and identified by botanist Professor J. H. Kim (Department of Life Science, Gachon University, Korea). A voucher specimen (no. Diab-2008-61) of the sample was deposited in the Herbarium of the Division of Korean Medicine Research, Korea Institute of Oriental Medicine (KIOM), Korea. The dried and ground plant material (3 kg) was extracted with EtOH (3 × 20 L) by maceration at room temperature for 3 days. The extracts were combined and concentrated *in vacuo* at 40°C to give an EtOH extract (390 g). The analysis of the quality and the standardization of LJE were performed by HPLC (data not shown). A major compound, epicatechin, as well as the crude extract were analyzed using a Shimadzu HPLC system (Shimadzu, Kyoto, Japan).

### 2.3. Metabolic and Morphological Analysis

When the rats reached 20 weeks of age, the blood glucose levels was measured using an automated analyzer (Wako, Japan). Glycated hemoglobin was determined by a commercial kit (Unimate HbA1c, Roche Diagnostics, Mannheim, Germany). Blood samples were collected from the tail vein after a 16 h fast. Individual mice were placed in metabolic cages to obtain 24 h urine collections, and the urinary protein excretion levels were measured. Renal cortexes were fixed in 10% formaldehyde and embedded in paraffin, and 4 *μ*m thick sections were prepared. The sections were stained with periodic acid-Schiff (PAS) reagent and hematoxylin as a counterstain.

### 2.4. *In Vitro* Assay of the Inhibition of AGE Formation and the Cross-Linking of AGEs with Proteins

According to a well-established method [14], the reaction mixture (bovine serum albumin [10 mg/mL; Sigma, St. Louis, MO, USA; 700 mL] in 50 mM phosphate buffer [pH 7.4] with 0.02% sodium azide) was added to 0.2 M fructose and glucose (100 mL). In screw-cap tubes (1.5 mL), the reaction mixture was then mixed with 200 mL of serial diluted LJE or aminoguanidine (AG) (Sigma). After the samples had been incubated at 37°C for 14 days, the fluorescent reaction products (200 mL) were transferred to 96-well plates and assayed using a spectrofluorimetric detector (BIO-TEK, Synergy HT, USA; Ex: 350, Em: 450 nm). The AGE assay was performed in triplicate. The concentration of each test sample resulting in 50% inhibition of the activity (IC_50_) was estimated from the least-squares regression line of the logarithmic concentration plotted against the remaining activity. For the AGE cross-linking inhibition assay [15], AGE-BSA (TransGenic Inc, Kobe, Japan) was incubated in either the presence or absence of LJE or AG collagen-coated 96-well plates. Collagen-AGE-BSA cross-linking was detected using a rabbit anti-BSA antibody, a horseradish peroxidase-linked goat anti-rabbit IgG antibody, and an H_2_O_2_ substrate containing the ABTS chromogen. The inhibition of collagen-AGE-BSA cross-linking was expressed as the percent decrease in optical density (OD = 410 nm). We calculated the IC_50_ concentration (*μ*g/mL) as the concentration at which collagen-AGE-BSA cross-linking was inhibited by 50%.

### 2.5. Immunohistochemistry

Renal sections were deparaffinized and hydrated by sequential immersion in xylene and graded alcohol solutions and then treated with normal serum from the same species that contained the secondary antibody to block nonspecific staining. The slides were incubated overnight at 4°C with mouse anti-AGE (1 : 200, TransGenic Inc, Kobe, Japan), rabbit anti-synaptopodin (Santa Cruz, CA, USA), and rabbit anti-nephrin (Abcam Inc, MA, USA). The slides were viewed by light microscopy (BX51, Olympus, Tokyo, Japan). For the detection of AGEs and nephrin, the sections were incubated with the appropriate regents from the Envision kit (DAKO, CA, USA) and were visualized using 3,3′-diaminobenzidine tetrahydrochloride (DAB system, Dako, CA, USA) and 3-amino-9-ethylcarbazole (ImmPACT AEC, VECTOR, CA, USA). For morphometric analysis, the number of positive cells or the area of positive signal per glomerulus in a total of 40 glomeruli was determined using Image J software (NIH, Bethesda, MD, USA).

### 2.6. SDS-PAGE and Western Blot Analysis

The isolated kidney cortexes were immersed in cell lysis buffer containing 1% protease inhibitor cocktail and homogenized using homogenization autolysis (Precellys, Bertin Technology, France). Renal tissues were separated by SDS—polyacrylamide gel electrophoresis and transferred to nitrocellulose membranes (Bio-Rad, CA, USA). The membranes were incubated with a rabbit anti-AGE antibody (1 : 200, TransGenic Inc, Kobe, Japan), and then the immune complexes were visualized using an enhanced chemiluminescence detection system (Amersham Bioscience, NJ, USA) and a Bio-Rad electrophoresis image analyzer (Bio-Rad, CA, USA).

### 2.7. RNA Extraction and Reverse Transcriptase PCR

Total RNA was extracted from renal cortexes using the Trizol reagent (Invitrogen Life Technologies, CA, USA) according to the manufacturer's instructions. The concentration of total RNA was calculated using the A260. Aliquots of total RNA (2 *μ*g each) from each sample were reverse transcribed into cDNA according to the instructions for the first strand cDNA synthesis kit (Bio-Rad, CA, USA). RNA (2 *μ*g) was used for reverse transcription polymerase chain reaction (PCR) using a thermal cycler (TAKARA, Otsu, Japan). The PCR products were run on a 1.5% agarose gel containing 0.5 *μ*g/mL ethidium bromide (EtBr). The quantification of the PCR products was performed using densitometry, and the relative mRNA levels were calculated and compared after normalization to beta-actin. The sequences were as follows: nephrin mRNA, sense 5′-CAACAGAAGACCACCAACCG-3′ and antisense 5′-CCTCCACTGGGACTGAAGGT-3′, and beta-actin mRNA, sense 5′-GGA AAG ACA ACG GAC AAA TC-3′ and 5′-GCTGCTGGTTTCCAAGTTCA-3′.

### 2.8. *In Situ* Hybridization Analysis

An 18-base-pair DNA fragment from the nephrin mRNA sequence (GenBank AF172256.1) was used as a probe. The sense and antisense primers were 5′-CAACAGAAGACCACCAACCG-3′ and 5′-CCTCCACTGGGACTGAAGGT-3′, respectively. The sense and antisense oligo probes were labeled with digoxigenin with a commercial kit (Boehringer Mannheim, IN, USA) according to the manufacturer's instructions. Renal tissues from each mouse were collected in 10% neutral buffered formalin, and after 1 or 2 days of fixation, they were dehydrated using graded alcohols and a xylene step and embedded in paraffin wax. Sections were then cut at 4 *μ*m, placed on Superfrost/Plus slides (Fisher Scientific, PA, USA), and stored at room temperature. Just before use, the sections were dewaxed in xylene and rehydrated in phosphate-buffered saline (PBS; pH 7.4, 0.01 M) for 5 min. Deproteinization was carried out in 0.2 N HCl for 20 min at room temperature. The tissues were then digested at 37°C for 20 min in 300 *μ*g/mL proteinase K (Gibco BRL, NY, USA) in PBS and fixed in 4% paraformaldehyde in PBS for 10 min. After being rinsed twice with PBS, the slides were acetylated in 300 mL of 0.1 mM triethanolamine-HCl buffer (pH 8.0) to which 0.75 mL of acetic anhydride (0.25%) had been added. After 5 min, a further 0.75 mL of acetic anhydride was added, and 5 min later, the slides were rinsed in 2x saline sodium citrate (SSC) (1x SSC contains 50 mM NaCl and 15 mM sodium citrate, pH 7.0). Hybridization was carried out overnight at 40°C. The digoxigenin-labeled probe (50 ng) was diluted in 50 *μ*L of the standard hybridization buffer, which consisted of 2x SSC containing 50% deionized formamide, 10 mg of salmon sperm DNA (Oncor, MD, USA), 0.02% sodium dodecyl sulfate (SDS), and 50% dextran sulfate solution (50% concentration). The slides were then heated for 10 min in a 95°C heating block and quenched on ice. Approximately 75 ng of digoxigenin-labeled probe was added to standard hybridization buffer (70 *μ*L), which was then layered over the section. The fluid was held in place by a coverslip (the edges of which were sealed with rubber cement), heated for 10 min in a 95°C heating block, and then quenched on ice. After hybridization overnight, the sections were thoroughly washed: twice in 4x SSC for 5 min at room temperature, twice in 2x SSC for 5 min at 40°C, twice in 2x SSC for 5 min at room temperature, twice in 0.2x SSC for 5 min at 40°C, twice in 0.2x SSC for 5 min, once in maleic acid buffer (100 mM maleic acid and 150 mM NaCl, pH 7.5) for 5 min, and once in 1x blocking reagent (Boehringer Mannheim, IN, USA) for 40 min at room temperature. For the detection of hybridization, sections were incubated with anti-digoxigenin conjugated with horse-radish peroxidase (Boehringer Mannheim, IN, USA). After three washes in buffer, substrate consisting by 3,3′-diaminobenzidine tetrahydrochloride (DAB system, Dako, CA, USA) was layered over the sections. The sections were counterstained with hematoxylin.

### 2.9. Statistical Analysis

Data are expressed as the mean ± SEM and were analyzed by one-way analysis of variance (ANOVA) followed by Tukey's multiple comparison test or by unpaired Student's *t*-test using GraphPad Prism 5.0 software (Graph pad, San Diego, CA, USA). Differences with a value of *P* < 0.05 were considered statistically significant.

## 3. Results

### 3.1. Inhibition of AGE Formation and Cross-Linking with Proteins

The results of the assays investigating the inhibitory effects on AGE formation and the cross-linking of AGEs with protein and the IC_50_ values of LJE are presented in Table 1. LJE exhibited 2.9-fold higher inhibitory activity against AGE formation than did aminoguanidine (IC_50_ = 62.40 *μ*g/mL), a well-known glycation inhibitor, after incubation at 37°C for 14 days (Table 1). In addition, the inhibitory effect of LJE on the cross-linking of AGE-BSA with collagen (IC_50_ = 17.38 *μ*g/mL) was much stronger, by 168-fold, than the effect of AG (IC_50_ = 2.92 mg/mL) (Table 1).

### 3.2. Body Weight and Blood Glucose

In db/db mice at 20 weeks of age, the body weight was greater than that of normal mice, and the body weight was lower in the mice in the LJE-250 group. The blood glucose was significantly increased in db/db mice (*P* < 0.01 versus normal). LJE induced only a minor decrease of levels of blood glucose. However, LJE failed to reduce HbA1c levels in db/db mice. MET resulted in a significant reduction in the blood glucose level (Table 2).

### 3.3. Morphology and Renal Function

**Figure 1:**
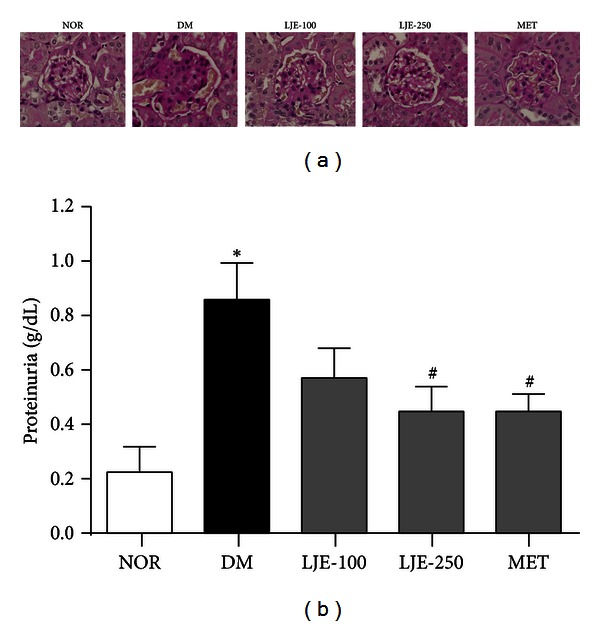
Renal histopathology and function. (a) Periodic acid-Schiff staining of glomeruli. ×400 magnification; (b) proteinuria in each group. NOR: normal mice; DM: diabetic db/db mice; LJE-100: DM treated with LJE (100 mg/kg); LJE-250: DM treated with LJE (250 mg/kg); MET: DM treated with metformin (350 mg/kg). All data are expressed as the mean ± SEM (*n* = 8). *P < 0.01 versus the NOR group; ^#^ *P* < 0.01 versus the DM group.

Mesangial matrix expansion is hallmark of diabetic nephropathy. At 20 weeks of age, db/db mice showed evidence of focal mesangial matrix expansion, and proteinuria was significantly increased in db/db mice. The mice in the LJE-250 and MET groups exhibited markedly ameliorated mesangial expansion and proteinuria compared with untreated db/db mice (Figures 1(a) and 1(b)).

### 3.4. Accumulation and Expression of AGEs

**Figure 2:**
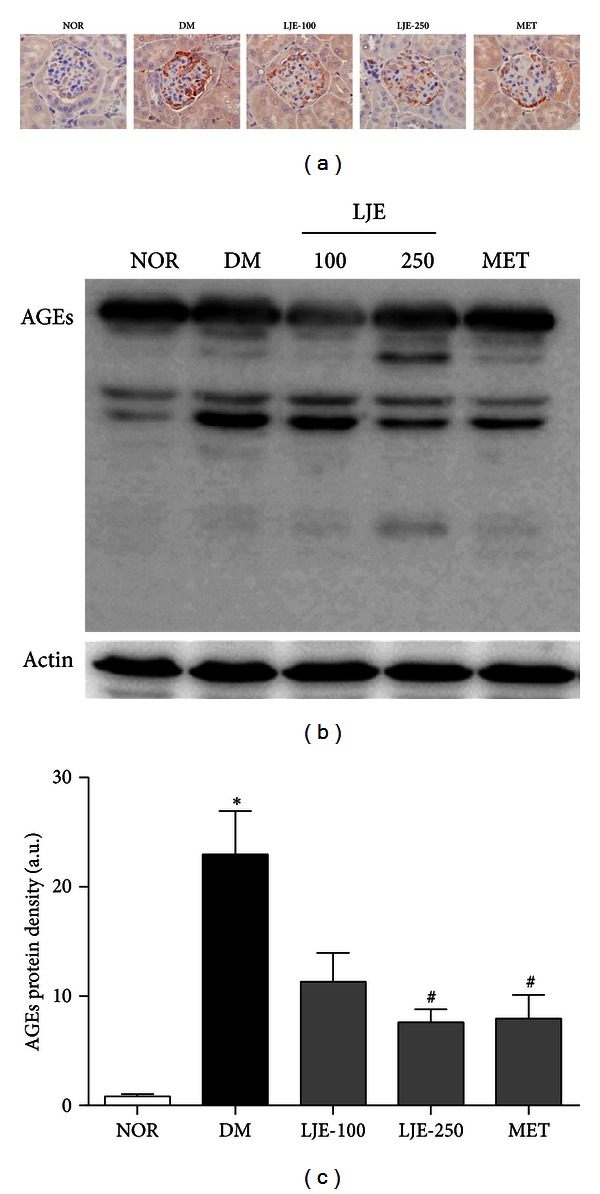
Effect of LJE on AGE accumulation in the renal glomeruli. (a) Immunohistochemical staining of AGEs. ×400 magnification. (b) AGE protein expression in each group. (c) Quantitative analysis of the AGE signals in histological sections. All data are expressed as the mean ± SEM (*n* = 8). *P < 0.01 versus the NOR group, ^#^ *P* < 0.01 versus the DM group.

In our *in vitro* study, LJE exhibited an inhibitory effect on AGE formation and the cross-linking of AGEs with proteins. To investigate the effect of LJE on AGE accumulation, we performed immunoassays and western blot analysis. Immunohistochemical staining of AGEs in glomeruli demonstrated a significant increase in db/db mice compared with normal mice. This increase was attenuated by LJE and MET (Figure 2(a)). In the western blot analysis, the AGE levels in the renal cortex were also markedly increased in db/db mice. LJE and MET reduced these diabetes-induced increases in AGE expression in a dose-dependent manner (Figures 2(b) and 2(c)).

### 3.5. Podocyte Loss in db/db Mice

**Figure 3:**
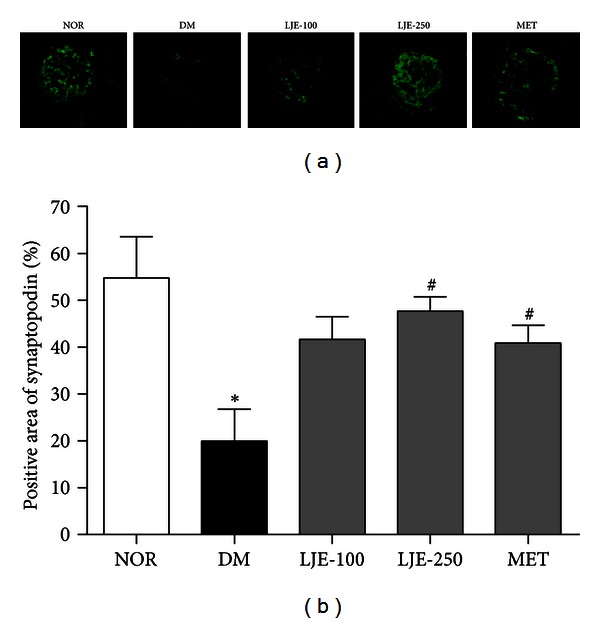
Effect of LJE on podocyte loss in the renal glomeruli. (a) Renal cortexes from mice were stained using a specific antibody for synaptopodin, which is a specific marker of podocytes. ×400 magnification. (b) Quantitative analysis of the podocyte signals in histological sections. All data are expressed as the mean ± SEM (*n* = 8). *P < 0.01 versus the NOR group, ^#^ *P* < 0.01 versus the DM group.

To investigate the effect of LJE on renal podocyte loss, an immunoassay was performed. The average number of podocytes per glomerulus was determined by counting cells and measuring the areas that were positively labeled by a podocyte marker, synaptopodin [16]. In db/db mice, the number of synaptopodin-positive cells was significantly lower than that in age-matched normal mice. Treatment with LJE-250 or MET visibly increased the number of positive cells and the positively stained areas in the renal glomeruli (Figures 3(a) and 3(b)).

### 3.6. Nephrin Expression in db/db Mice

**Figure 4:**
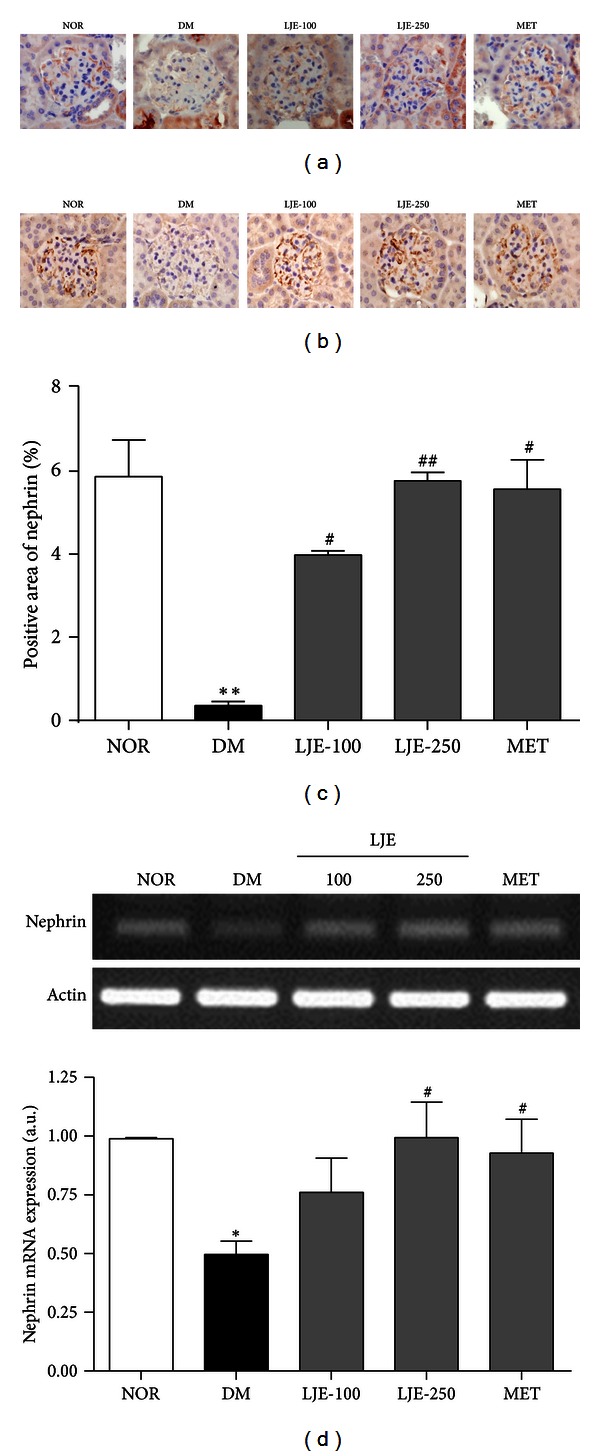
Effect of LJE on nephrin expression in the renal glomeruli. (a) Immunohistochemical signal for nephrin. (b) In situ hybridization of nephrin mRNA. ×400 magnification. (c) Quantitative analysis of the nephrin protein signals in histological sections. (d) RT-PCR using a nephrin-specific primer. All data are expressed as the mean ± SEM (*n* = 8). *P < 0.05 versus the NOR group, **P < 0.01 versus the NOR group, ^#^ *P* < 0.05 versus the DM group, ^##^ *P* < 0.01 versus the DM group.

Nephrin is a novel podocyte-specific protein that localizes to the slit diaphragm [17]. We investigated the expression of the protein and RNA levels of nephrin in the renal cortex by performing an immunoassay, in situ hybridization and RT-PCR. Nephrin in normal mice was strongly expressed in renal glomeruli. In untreated db/db mice, the expression of nephrin was weaker than that in the normal mice. However, the staining of nephrin in the renal glomeruli of LJE- and MET-treated db/db mice showed much higher nephrin levels than those of the untreated db/db mice (Figure 4(a)). To confirm the results obtained from immunohistochemistry, we performed an in situ hybridization analysis and RT-PCR. Untreated db/db mice had significantly lower levels of nephrin than normal mice did, and the decreased nephrin expression in the glomeruli of diabetic mice was restored by treatment with LJE and MET (Figures 4(b), 4(c) and 4(d)). These results suggest that LJE prevents nephrin loss in the context of diabetic nephropathy by stabilizing slit diaphragm proteins.

## 4. Discussion

The results of this study indicate that the use of LJE, an indigenous plant in Korea, may reduce the severity of diabetic renal complication. Our study showed that LJE exhibits stronger inhibitory activity against AGE formation and the cross-linking of AGEs with proteins than AG *in vitro. *Our results also showed that LJE, an herbal AGE inhibitor, reduced the development of diabetic nephropathy in type 2 diabetic db/db mice. Our current study demonstrated that LJE-treated diabetic mice showed significant improvements in renal function markers such as proteinuria. In addition, LJE prevented AGE accumulation in diabetic kidney and reduced podocyte loss and the decreased expression of nephrin, which is a slit diagram protein that plays an important role in podocyte function. LJE induced only a minor decrease of levels of blood glucose, but this difference was not statistically significant. In addition, LJE failed to reduce HbA1c levels in db/db mice. LJE has significant effects on any parameters of renal structure and function without the strong reduction of blood glucose. These findings suggest that even in hyperglycemia, it is possible to attenuate diabetic nephropathy by LJE.

We used an animal model of type 2 diabetes to investigate diabetic nephropathy. The db/db mouse develops glomerular hypertrophy, significant proteinuria, glomerulosclerosis, and insulin resistance with abnormalities that resemble the course of type 2 diabetic nephropathy in humans [18, 19]. In db/db mice, AGEs induce mesangial expansion and proteinuria [20]. Although various initiators of diabetic nephropathy have been proposed, including glycation, the polyol pathway and oxidative stress, one of the major consequences of hyperglycemia is the formation of AGEs. The formation of AGEs in renal tissue is closely correlated with the development of diabetic nephropathy [21, 22]. The irreversible formation of AGEs affects proteins and lipids such as hemoglobin, collagen, and lipoprotein and causes damage to the kidneys, eyes, and blood vessels [23]. Moreover, AGEs induce the apoptosis of cultured murine podocytes and have been proposed to be a potential pathogenic factor of podocyte loss [5]. Podocytes appear to be sensitive targets of the action of AGEs because they express the receptor for AGEs (RAGE) [24]. Podocyte damage has been demonstrated to correlate with worsening proteinuria and albuminuria [25]. Consistent with this interpretation, our results showed that the expression of synaptopodin, a podocyte marker [26], was lower in the AGE-accumulated glomeruli in db/db mice with severe proteinuria. However, treatment with LJE ameliorated the enhanced diabetic nephropathy in db/db mice.

There is considerable interest in compounds that inhibit AGE formation because of the therapeutic potential of such compounds [27]. Several natural and synthetic compounds have been proposed as inhibitors of AGE formation. AGE inhibitors, such as AG and LR-90, attenuate mesangial expansion and proteinuria in diabetic animal models [28–30]. Our previous studies showed that KIOM-79, a natural AGE inhibitor, prevented the decrease in renal function due to podocyte damage in type 2 diabetes animal models [31]. Epicatechin, a marker compound isolated from *L. japonica*, is a chemical standard for LJE and has a preventive effect on the formation of AGEs [32, 33]. Therefore, the ability of LJE to protect against renal damage may be due to the effect of this compound.

In this study, we also found that the treatment of db/db mice with LJE ameliorated the podocyte loss and the decreased nephrin mRNA and protein levels in AGE-accumulated glomeruli in the kidney. The slit diaphragms (SDs) formed by podocytic processes are important components of the glomerular filtration barrier. Slit diaphragms are zipper-like membranous electrosense structures that are formed by the linkage of podocytic processes in a zigzag manner. Nephrin is a single-spanning transmembrane Ig superfamily protein and an integral component of the podocyte slit diaphragm (SD), a structure that is critical to the glomerular selectivity filter [34]. The SD is the widest part of the podocyte structure, and the deletion of these structures may relax the podocytes, leading to their disappearance, thus disrupting the filtration barrier and inducing proteinuria [8, 35]. Recently, it has been reported that nephrin deficiency is due to the loss of podocyte function [8, 36].

In this study, we also compared the effect of LJE with that of metformin. Metformin has been widely used for treating type 2 diabetes without stimulating insulin production [37]. Metformin significantly decreases the urine albumin excretion rate in patients with type 2 diabetes [38]. The previous studies also have shown that metformin is a potent inhibitor of AGE formation [39]. Our study showed that LJE treatment afforded a level of inhibitory effects similar to that of metformin. These observations demonstrate that LJE has potential clinical uses to prevent diabetic nephropathy.

Although a major chemical standard of LJE is epicatechin, the most active compound of LJE needs to be identified. Nevertheless, our study still sheds light on a potential treatment strategy for diabetic nephropathy. In this study, LJE suppressed the generation of AGEs, which are an indicator of diabetic complications, and enhanced the stabilization of podocytes in the kidneys of db/db mice. In addition, the expression of nephrin, a slit diagram protein that plays an important role in podocyte function, was restored. Although the pathogenesis of diabetic nephropathy is multifactorial, microinflammation and subsequent extracellular matrix expansion are common pathways for the progression of diabetic nephropathy [40]. In previous study, LJE also had an anti-inflammatory effect [12, 13]. Therefore, LJE is likely exerting some of its effects via improvement renal inflammatory stress. It could be reasonable to assume that the renoprotective effect of LJE might be at least partly, attributed to its influence on AGEs formation and inflammatory status. LJE could be beneficial agent by protecting against renal complications of diabetes.

## Figures and Tables

**Table 1:** Inhibitory effect of LJE on AGE formation and cross-linking *in vitro*.

	AGE formation	AGE cross-links with protein
LJE (IC_50_)	21.21 ± 0.43 *μ*g/mL	17.34 *μ*g/mL
Aminoguanidine	62.40 *μ*g/mL	2.92 ± 0.39 mg/mL

Inhibitory activity was expressed as the mean ± SD of triplicate experiments. The IC_50_ values were calculated from regression lines using six different concentrations in triplicate experiments.

**Table 2:** Metabolic and physical parameters.

	NOR	DM	LJE-100	LJE-250	MET
Body weight (g)	26.89 ± 1.15	34.19 ± 6.51*	31.39 ± 3.23	30.86 ± 4.08^#^	32.65 ± 0.55
Blood glucose (mg/dL)	120.2 ± 35.74	774.0 ± 14.53*	694.9 ± 176.4	586.1 ± 216.8	674.0 ± 52.3^#^
HbA1c (%)	3.56 ± 0.07	7.31 ± 0.81*	7.91 ± 0.85	7.34 ± 1.31	6.23 ± 0.48

NOR: normal mice; DM: diabetic db/db mice; LJE-100: DM treated with LJE (100 mg/kg); LJE-250: DM treated with LJE (250 mg/kg); MET: DM treated with metformin (350 mg/kg). All data are expressed as the mean ± SEM. *P < 0.01 versus the NOR group; ^#^ *P* < 0.01 versus the DM group.
